# Inspiring health worker motivation with supportive supervision: a survey of lady health supervisor motivating factors in rural Pakistan

**DOI:** 10.1186/s12913-016-1641-x

**Published:** 2016-08-17

**Authors:** Fauziah Rabbani, Leah Shipton, Wafa Aftab, Kashif Sangrasi, Shagufta Perveen, Aysha Zahidie

**Affiliations:** Department of Community Health Sciences, Aga Khan University, Stadium Road, P.O. Box 3500, Karachi, 74 800 Pakistan

**Keywords:** Community health worker, Motivation, Supportive supervision, Health system, Implementation research, Lady health supervisor, Lady health worker, Pakistan

## Abstract

**Background:**

Community health worker motivation is an important consideration for improving performance and addressing maternal, newborn, and child health in low and middle-income countries. Therefore, identifying health system interventions that address motivating factors in resource-strained settings is essential. This study is part of a larger implementation research project called Nigraan, which is intervening on supportive supervision in the Lady Health Worker Programme to improve community case management of pneumonia and diarrhea in rural Pakistan. This study explored the motivation of Lady Health Supervisors, a cadre of community health workers, with particular attention to their views on supportive supervision.

**Methods:**

Twenty-nine lady health supervisors enrolled in Nigraan completed open-ended structured surveys with questions exploring factors that affect their motivation. Thematic analysis was conducted using a conceptual framework categorizing motivating factors at individual, community, and health system levels.

**Results:**

Supportive supervision, recognition, training, logistics, and salaries are community and health system motivating factors for lady health supervisors. Lady health supervisors are motivated by both their role in providing supportive supervision to lady health workers and by the supervisory support received from their coordinators and managers. Family support, autonomy, and altruism are individual level motivating factors.

**Conclusions:**

Health system factors, including supportive supervision, are crucial to improving lady health supervisor motivation. As health worker motivation influences their performance, evaluating the impact of health system interventions on community health worker motivation is important to improving the effectiveness of community health worker programs.

**Electronic supplementary material:**

The online version of this article (doi:10.1186/s12913-016-1641-x) contains supplementary material, which is available to authorized users.

## Background

Large-scale, functional community health worker programs (CHW-P) make important strides to counteract health workforce shortages in low and middle-income countries (LMICs) by extending primary healthcare to rural and underserved communities [1–3]. Community health workers (CHWs) in these programs are often affiliated with government healthcare systems and receive on-the-job training [1–4]. They are residing community members knowledgeable of community norms and trained to address community health concerns including family planning, infectious disease, and nutrition through healthcare and education [1, 2].

CHW motivation, often categorized into individual, community, and health system levels [5, 6], is important to the efficiency of healthcare delivery in LMICs [1, 7, 8]. This is because health worker motivation considerably influences performance and productivity as reflected by health worker commitment and readiness to use their knowledge and skills to fulfill their responsibilities [8, 9]. Logistic considerations, salaries and financial incentives, training, empowerment, recognition, and altruism are CHW motivating factors identified in the literature [5, 10–16]. For example, during in-depth interviews in Bangladesh, CHWs reported being motivated to continue their work because financial incentives supported their household expenses and independence [17]. In interviews and surveys conducted in Mexico and Uganda, CHWs identified training as motivating because they used the new knowledge to help their families and communities [10, 18, 19]. CHWs from various LMICs have described increased social status as healthcare providers and educators to be a motivating factor [10–14, 18, 20].

In Pakistan, Lady Health Workers (LHWs) of the Lady Health Worker Programme (LHW-P) are assigned to underserved and rural communities without proximate health centers [21–23]. The LHW-P covers approximately 60 % of Pakistan’s population and was implemented in 1993-1994 with the aim of training 100, 000 LHWs on basic healthcare by 2005. According to a 2006 case study report, a total of 96, 000 LHWs have been trained [24]. Each LHW serves a geographic area covering 100–150 households and approximately 1,000 people. LHWs are preferably married women aged 18–45 with at least eight years of schooling and approval from the community [21, 23, 25]. They are trained to provide treatment and health education for maternal, newborn, and child health (MNCH). Lady Health Supervisors (LHSs) are another cadre of CHWs working in the LHW-P who are responsible for directly managing 25–30 LHWs. LHSs make monthly visits to each LHW to supervise their community case management (CCM) skills during visits to community households. LHSs are expected to provide supportive supervision to LHWs [21, 25], which aims to improve LHW performance and quality of CCM through active monitoring, constructive feedback cycles, training, problem solving, and open communication [21, 22, 25–27]. LHSs have at least eight years of education, previous work experience as a Lady Health Visitor or LHW, and reside within the community. They report to the Assistant District Coordinator (ADC) of the LHW-P. LHWs typically work alone under the guidance of their LHS, but attend monthly meetings with LHSs and LHWs in their district to discuss health progress and issues [21].

Despite the LHW-P efforts, under five child mortality from pneumonia and diarrhea has remained relatively stagnant in Pakistan [21, 25]. The LHW-P weaknesses include inconsistent salaries, job insecurity, overworked LHSs and LHWs, and inadequate supply of medicines [23]. Similar to other national CHW-P [3, 14, 16, 28, 29], the LHW-P supervision structures require improvement [1, 23]. For example, supervision structures breakdown when LHSs do not consistently make supervisory visits to monitor and evaluate LHW performance in the community [21]. This is of concern because supervision is crucial to CHW-P functioning [3, 30], and when adequately implemented, supportive supervision structures contribute to CHW performance and productivity by fostering motivation and a positive work environment [8, 31, 32]. Effective supervision strategies require well-defined supervisor responsibilities, effective training, and an emphasis on supportive communication approaches [1, 30, 33, 34].

Aware of supervision as an area of improvement for the LHW-P, Nigraan, an implementation research project, intervened on the LHW-P supportive supervision structures to improve CCM of pneumonia and diarrhea in a district of rural Pakistan [21]. This study is part of Nigraan, and aimed to explore LHS motivating factors, with particular interest in how their views on supportive supervision contribute to the literature and inform on ways that CHW-P can facilitate motivating supervisory relationships.

## Methods

### Overview of Nigraan

Nigraan (meaning supervisor in Urdu) is an implementation research project using a cluster-randomized design to assess the impact of strengthening supportive supervision and clinical mentorship in the LHW-P on LHSs, LHWs, and community caregivers (mothers of children under five) [21]. The Nigraan intervention worked with the existing health system and was two-pronged. First, although both arms received LHW-P curriculum refresher training, the intervention arm received an enhanced course in supportive supervision and clinical mentorship using interactive teaching pedagogies such as audiovisual aids and role-playing. Second, supervisory tools were introduced to supplement the LHW-P management information system (MIS). The LHW-P MIS gathers case management data at the community level and transfers it to district, provincial, and national offices, in order to assess LHW performance [22]. The supervisory tools included a LHW-P supervisory checklist modified to encourage direct CCM monitoring, a quality of case management sheet to track progress and reveal gaps in CCM practices, and a feedback card for LHSs to provide written assessments for LHWs. To facilitate the intervention, Nigraan installed a surveillance system using simple mobile phones to improve communication and coordination between LHSs and LHWs regarding case detection, tracking, management, and follow-up. LHSs were provided simple mobile phones and LHWs were given a small communication allowance, so implementation costs remained low [35]. Nigraan also scheduled regular meetings with each arm to discuss project progress and recognize LHS efforts with certificates of participation and performance.

Nigraan took place in District Badin of Sindh, Pakistan. The district was selected because it is representative of LHW-P infrastructure and functionality at provincial and national levels [21]. A total of 34 LHSs and 170 LHWs, evenly and randomly distributed into intervention and control arms, participated in Nigraan.

### Study setting and participants

Participants in this study were LHSs working for the LHW-P and enrolled in Nigraan. Thirty-four LHSs were invited to attend one of two progress meetings, during which the survey was administered. Twenty-nine LHSs were able to attend one of the meetings and complete the survey, while five LHSs did not attend due to personal or family reasons. The first 15 LHSs were surveyed in January 2015 and the next 14 LHSs were surveyed in April 2015.

### Data collection and analysis

A structured survey of ten open-ended questions was designed to gather LHS perceptions of motivation. These questions asked when LHSs feel happy and supported, ways they give and receive motivation, and benefits and difficulties of working for the LHW-P. The survey template in English is available as Additional file 1. The survey instrument was developed based on information obtained from previous Nigraan qualitative inquiry with LHSs and LHWs as well as a literature review of CHW motivation in LMICs. The survey was written in English and translated into Urdu.

Each data collection session took an average of one hour and each group of LHSs were surveyed in the same room so that Nigraan team members were available to provide clarification of questions if necessary. The surveys did not ask for LHS names or identification, so LHSs were assured of their anonymity and encouraged to respond honestly to each question. All LHSs were able to read the surveys written in Urdu, but wrote their responses in Sindhi or Urdu. Two members of the Nigraan team, who are fluent in English, Sindhi, and Urdu, translated survey responses into English.

Nvivo software Version 10.2 was used to analyze the survey responses, which were read twice before coding began. Once familiarized with the data, researchers coded iteratively to categorize data into themes. Codes were informed by Gopalan and colleagues conceptual framework that categorizes CHW motivating factors into individual, community, and health systems levels [6]. This organization of CHW motivation is well recognized in the literature and illustrates that CHWs are motivated by various levels of society [5, 6, 14].

## Results

**Fig. 1:**
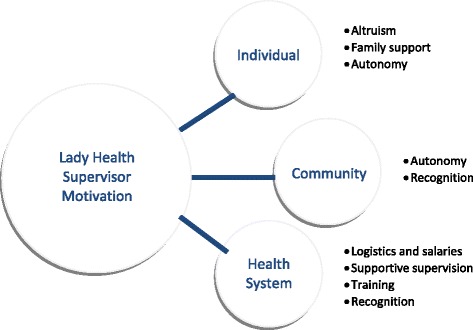
Individual, community, and health system level motivating factors of Lady Health Supervisors

LHS survey responses were categorized into motivating factors at individual, community, and health system levels, which are illustrated in Fig. 1.

### Community and health system level motivation

Recognition, supportive supervision, training, logistics and salaries are factors that motivate LHSs at the community and health system level.

#### Recognition

LHSs believe they have built a positive reputation in communities through knowledge and skill contributions. Therefore, many LHSs feel motivated when their work is appreciated and respected by the community members they support.

*[We feel happy] when the community admires our work related to diarrhea, pneumonia, and vaccination in the field.* (LHS 7)

This recognition is also important from members of the health system, such as coordinators, managers, and fellow LHWs.

*I feel happy when my LHWs praise me in front of others.* (LHS 28)

*[We feel happy] when we are praised by the Assistant District Coordinator or other upper staff.* (LHS 27)

LHSs enjoy being recognized publically for their work, whether from the community or staff in the health system. Some LHSs describe the certificates of participation and performance presented at Nigraan progress meetings as helpful to their motivation and a way to also motivate LHWs.

*I feel happy when I work in the field and share my knowledge with the community. We also get certificates and financial assistance that is very helpful.* (LHS 26)

*The hardworking LHWs should be praised and can [also] be given honorary certificates for good performance.* (LHS 2)

#### Supportive supervision

LHSs are motivated by the supervision they receive from coordinators and managers in the LHW-P and by their role in supervising LHWs. LHSs appreciate guidance from the ADC, but they also want to be supported by LHW-P managers and fellow health professionals.

*[I am motivated to work hard] when our program managers listen to our problems and try to solve them.* (LHS 23)

*For improvement in our work quality someone should check our work and encourage us.* (LHS 5)

LHSs are proud of their supervisory skills and are encouraged when their LHWs and fellow LHSs provide good care to communities. It is important to LHSs that they provide meaningful supervision to LHWs by exercising patience and support when teaching.

*We motivate the LHWs by praising her hard work in the community.* (LHS 16)

*I feel good when LHWs do good work by treating the community well and [when they are] appreciated by the community.* (LHS 18)

*As an LHS I treat my LHWs with respect and if they don’t understand anything I train them and I do my job properly being their supervisor.* (LHS 28)

*[I am motivated when] I have complete knowledge, skills, and coordination with LHWs to give regular feedback for supportive supervision.* (LHS 2)

In return, LHSs want to feel respected by the LHWs that they supervise. When supervisory relationships are positive, LHSs believe they are able to improve the standard of CCM provided by their LHWs.

*When an LHW respects us and follows [our] advice then we feel motivated to work with them and improve their understanding.* (LHS 1)

*If we perform our job properly, LHWs will also do work satisfactory.* (LHS 6)

#### Training

Training opportunities motivated LHSs to join the LHW-P because they wanted to gain knowledge and skills to help their families and communities. LHSs also emphasize that regular training is important to feeling confident in their work because community members expect them to be knowledgeable of health issues.

*When we go to the community and share our information regarding maternal and child health issues, and when we see them again, they expect us to share more knowledge and that makes us feel happy.* (LHS 24)

*We don’t get official trainings from our program. When we do get trainings our knowledge is going to increase and then we can do our job properly.* (LHS 28)

LHSs are especially motivated when they see their knowledge improving lives.

*When I go to community with my LHW to solve their problems we motivate the caregivers and give awareness to the community. The community listens to us carefully and asks questions. When a mother or child has any danger sign and we refer him/her to the health facility, then we feel very happy that we saved someone’s life by creating awareness.* (LHS 22)

#### Logistics and salaries

LHSs are demotivated when medical supplies and salary allowances are delayed or undelivered. Working without transportation or the fuel necessary to fulfill their community visits to supervise LHWs is an additional reason LHSs become unmotivated. The lack of fuel, medicines, and salary interferes with LHS job satisfaction because they cannot perform their duties properly.

*Our pay is meager, that’s why we feel difficulty in performing our job.* (LHS 6)

*I’m tired of working due to salary and petrol issues. We are so dissatisfied that sometimes we feel like quitting our job.* (LHS 14)

*[It is difficult] when we don’t get salary for three months, fuel for one year, and no medicines are available for field work.* (LHS 25)

Delayed supply of medicines is problematic because community members view LHSs more favorably when they provide medicines. LHSs are aware that when they arrive consistently without medicines, the community is disappointed.

*The community can benefit and trust us more if we have medicines available with us.* (LHS 25)

The delayed or minimal salaries are particularly demotivating for LHSs. However, while many LHSs describe salary delays as a main reason they would resign from the LHW-P, other LHSs were adamant that the income was too important to their household for them to quit.

*When I don’t receive pay for three months, I sometimes feel I should just quit this job.* (LHS 15)

*I can’t think of leaving this job, as it’s the only source of income for me.* (LHS 21)

### Individual level motivation

Family support, altruism, and autonomy comprised individual level motivation.

#### Family support

LHSs describe the approval or disapproval of their families as influential to their motivation and their ability to continue working with the LHW-P. LHSs worry that their husbands or other family members would demand they resign from the LHW-P. Other LHSs who feel supported by their family members find it easier to fulfill their work responsibilities.

*If I get any resistance from my family, I will quit this job. But I pray to God that this doesn’t ever happen, as I won’t get another job.* (LHS 19)

*We feel motivated when our family supports us and then we can do our job properly.* (LHS 16)

#### Altruism

LHSs are motivated by their ability to use their knowledge and skills to save lives and improve child health in their communities. LHSs feel morally obligated to elevate community awareness of health issues, such as pneumonia and diarrhea. This altruism motivates LHSs to continue working despite the difficulties they face.

*The thought that we can save a child’s life by going to the field and working with the community makes us feel happy.* (LHS 16)

*Despite the problems of irregular supplies we consider it a social work and serve the community, which does not let us think of leaving this job.* (LHS 2)

#### Autonomy

LHSs feel more independent in their personal lives because their work with the LHW-P provides them a salary to fund their children’s education and contribute to household affairs.

*We get a salary that we can use independently to solve our problems.* (LHS 28)

*When we get our salary we can independently make decisions to use for our children’s education.* (LHS 26)

It is difficult for LHSs to find work because they live in rural communities, so they are appreciative of the opportunity to work with the LHW-P. LHSs are confident in their knowledge and skills and community role, and show competence in understanding the communities that they serve.

*We like this work a lot and we are part of a successful profession now.* (LHS 20)

*When we go to the field, we know the different practices of people and the problems of community, based on which we can make decisions.* (LHS 7)

## Discussion

In this study, LHSs were surveyed to learn factors that motivate their work with the LHW-P. LHS motivating factors were analyzed into themes at the individual, community, and health system levels. Overall, these findings align with similar research on CHW motivation in LMICs. Motivating factors at the community and health system levels are supportive supervision, recognition, training, logistics, and salaries. At the individual level the motivating factors are family support, autonomy, and altruism. In this study, the role of supportive supervision in motivating LHSs was of particular interest, and was described by LHSs as an important motivating factor at the health system level. As this study was part of Nigraan, a larger health system implementation research project, we interpreted these findings in terms of how health system programs, such as the LHW-P, can use and respond to these motivating factors.

LHSs in this study are motivated by the support they receive from LHW-P coordinators and managers as well as the supervision they provide to LHWs. As recipients of supervision, LHSs desire appreciative and encouraging support from the ADC and other LHW-P management. They also seek respect from the LHWs they supervise and are motivated when they perform their duties well in the community. Supervision as a motivating factor is recognized in other studies [14, 16, 20, 35], but primarily from the vantage point of supervision recipients comparable to LHWs of the LHW-P who are not in supervisory positions. LHSs are CHWs in a supervisory role, so these findings add unique insight on ways supervisors are motivated. CHWs from studies in Malawi, Tanzania, and Zambia do not comment on supportive supervision style specifically, but are receptive to the tone and presence of supervision, candidly describing its motivating influence [14, 16, 20]. In Tanzania and Zambia, CHWs were motivated by supervision that facilitated their learning and skill development [14, 16]. However, many CHWs were demotivated by supervisors unwilling to teach, problem solve, or support their role in the community [14, 16, 20]. LHSs are in the unique position, as compared to other CHWs, of receiving supervision and providing supportive supervision. As recipients of supervision, LHSs are similar to CHWs in these studies, in that they are motivated when they receive support from their coordinators and managers that is encouraging and attentive to their work problems. However, in their position as providers of supportive supervision LHSs are motivated by their ability to motivate LHWs to perform well in their communities. LHSs try to motivate LHSs by giving them respect, offering encouragement, and providing advice and training. In return, LHSs are motivated when their LHWs give them respect, improve their performance, and are engaged with and accountable to the communities they serve. In order to better understand positive supervisory relationships in the context of CHW-P, perceptions of the recipients of supportive supervision, such as LHWs, should be explored for comparison with supervisor perspectives.

Training opportunities, such as those provided by Nigraan also motivated LHSs in this study. Similar to CHWs in other LMICs [14, 17–19, 36], LHSs desired training to gain knowledge and skills that can help their families and communities. For example, CHWs in Malawi gained confidence from training sessions because it strengthened their community contribution [20]. In the case of Nigraan, training in supervision and CCM of pneumonia and diarrhea motivated LHSs. Research into CHW perceptions of various teaching pedagogies may identify CHW-P training strategies most effective for knowledge uptake and transfer.

As with CHWs from sub-Saharan Africa and South Asia [11, 12, 14, 17, 18, 20], LHSs in this study were also motivated by gains in community respect and prestige sourced from their position. Additionally, LHSs were motivated by updates on Nigraan’s research progress and certificates of performance and participation presented at regularly scheduled meetings. This finding illustrates that gestures of appreciation and recognition from the LHW-P coordinators and managers have potential to motivate LHSs. Furthermore, as with CHWs in Zambia [16], LHSs desire career advancement opportunities. Therefore, creating avenues of promotion or recognition may be an effective way to retain LHSs and motivate their work with the LHW-P.

LHSs conveyed the importance of family approval in allowing their work with the LHW-P to continue. LHSs were fearful that their families would demand they resign from the LHW-P. Although they did not provide explanations for this disapproval, CHWs in regional neighbor Bangladesh described safety concerns, female mobility, and gender norms as reasons their families disapproved of their work [15, 17]. For CHWs in Tanzania, family support with domestic and farm work was crucial to their ability to work [14]. Reasons for and influence of family approval or disapproval can vary regionally, therefore attention should be given to ways CHW-P can respond to the concerns of family members so they support their female relatives working as CHWs.

LHSs in this study share the financial and logistical frustrations of CHWs in South Asia and sub-Saharan Africa, in that they feel underpaid and undersupplied by the LHW-P [11, 14, 16, 17]. In-depth interviews and focus group discussions with CHWs from rural Tanzania and Ghana reported that inadequate salaries are common reasons for resignation or dissatisfaction [11, 14]. In Bangladesh, CHWs signaled unmet financial expectations as demotivating and disappointing [12, 13, 15]. This point is further illuminated by CHWs deployed as part of a national strategy in Zambia, who reported being demotivated because stipends promised by the government were only partially delivered, or not at all [16]. As with LHSs, Zambian CHWs described how limited medical supplies discredited their work in communities. These findings are unsurprising [23], but nonetheless highlight important areas of improvement in the LHW-P.

### Limitations

Although this study did not endeavor to generalize, a larger sample size would have gathered greater depth and understanding of LHS perceptions. However, this study surveyed LHSs in a district that closely reflects provincial and national level program infrastructure and functionality, so it is perceived that these findings would resonate with LHSs in other districts of the LHW-P. During data collection, surveys were administered to a group of LHSs, so it is possible that discussion amongst LHSs influenced their responses. However, Nigraan team members were present during the survey to keep discussion to a minimum and assure LHSs that their individual responses were valued, anonymous, and not scored. The rapport built between the Nigraan team and the LHS participants during the ongoing larger project was also important to LHSs feeling comfortable responding honestly to the survey.

## Conclusions

LHSs in this study added insight to the complex portfolio of motivating and demotivating factors experienced while working for the LHW-P in Pakistan. Motivating factors clearly positioned at individual, community, and health system levels of influence were reflective of CHWs in other LMICs. The role of supervision for LHSs, either as recipients from upper level staff or as supervisors to LHWs, was an important finding that exposed the potential for positive supervisory relationships to improve motivation and performance in the LHW-P. This focus on supervisory relationships can be explored further in other CHW-P. Recognition, training, and logistics and salaries were other motivating factors identified by LHSs that can be directly influenced by CHW-P policies and resources, and should be a priority for improving CHW performance. As health system factors are integral to CHWs motivation, evaluating health system interventions for their impact on CHW motivation will be important to improving their performance and CHW-P effectiveness.

## Additional file

Additional file 1: Lady Health Supervisor Motivation Survey Template (English). (DOCX 15 kb)

## Abbreviations

ADC: Assistant District Coordinator
CCM: Community Case Management
CHW: Community Health Worker
CHW-P: Community Health Worker Program
LHS: Lady Health Supervisor
LHW: Lady Health Worker
LHW-P: Lady Health Worker Programme
LMIC: Low and Middle-Income Countries
MIS: Management Information System
MNCH: Maternal, Newborn, and Child Health
