# Supplementary material for: Inspiring health worker motivation with supportive supervision: a survey of lady health supervisor motivating factors in rural Pakistan
Source: BMC Health Serv Res. 2016 Aug 17;16:397. doi: 10.1186/s12913-016-1641-x (PMC4989332; doi:10.1186/s12913-016-1641-x)
Supplement: Additional file 1: — Lady Health Supervisor Motivation Survey Template (English). (DOCX 15 kb) [file 12913_2016_1641_MOESM1_ESM.docx]

**Lady Health Supervisor Motivation Survey Template (English)**

Participants: Lady Health Supervisors

Time: 45-75 minutes

Medium of Communication: Urdu/Sindhi

Dear Lady Health Supervisors, we would like to ask you questions about your work with the Lady Health Worker Programme. Kindly answer the following questions to the best of your ability.

1. When do you feel happy as a lady health supervisor?
2. What motivates you to work hard as a lady health supervisor?
3. How do you feel being a lady health supervisor has benefited you?
4. How does your job make you financially empowered?
5. What are important financial and material gains as a lady health supervisor?
6. What are you strengths as a lady health supervisor?
7. How do you encourage your lady health workers to work hard?
8. Do you feel supported at work? If yes, who/what supports you? If no, what would make you feel more supported?
9. What would be some reasons that would make you consider leaving your position as a lady health supervisor?
10. What makes your work difficult to do?
